# Sex-Specific Differences in Lipid and Glucose Metabolism

**DOI:** 10.3389/fendo.2014.00241

**Published:** 2015-01-19

**Authors:** Oleg Varlamov, Cynthia L. Bethea, Charles T. Roberts

**Affiliations:** ^1^Division of Diabetes, Obesity, and Metabolism, Oregon National Primate Research Center, Beaverton, OR, USA; ^2^Division of Developmental and Reproductive Sciences, Oregon National Primate Research Center, Beaverton, OR, USA; ^3^Department of Behavioral Neuroscience, Oregon Health and Science University, Portland, OR, USA; ^4^Department of Medicine, Oregon Health and Science University, Portland, OR, USA

**Keywords:** adipose tissue, androgens, estrogens, fatty acid, insulin sensitivity, obesity, sex differences

## Abstract

Energy metabolism in humans is tuned to distinct sex-specific functions that potentially reflect the unique requirements in females for gestation and lactation, whereas male metabolism may represent a default state. These differences are the consequence of the action of sex chromosomes and sex-specific hormones, including estrogens and progesterone in females and androgens in males. In humans, sex-specific specialization is associated with distinct body-fat distribution and energy substrate-utilization patterns; i.e., females store more lipids and have higher whole-body insulin sensitivity than males, while males tend to oxidize more lipids than females. These patterns are influenced by the menstrual phase in females, and by nutritional status and exercise intensity in both sexes. This minireview focuses on sex-specific mechanisms in lipid and glucose metabolism and their regulation by sex hormones, with a primary emphasis on studies in humans and the most relevant pre-clinical model of human physiology, non-human primates.

## Introduction

While the role of sex in biology is undisputed, its consideration in research has been the subject of much recent debate. Although initial concerns were raised with the underrepresentation of women in clinical studies (1, 2), this issue also applies to pre-clinical research in many fields (3, 4). More recently, the National Institutes of Health is considering a proposal to require representation of both sexes in animal as well as cell-based studies (5, 6); in the latter instance, the effect of the sex of the source tissue, especially in the case of established cell lines, is not often considered (7). In parallel, a growing number of biomedical research journals are requiring specific clarification of the sex of animals and of the source tissue of cells used in published studies. In this context, it is timely to consider the sex-specific aspects of glucose and lipid metabolism in light of the disruption of these regulatory mechanisms in clinical pathologies such as metabolic syndrome, obesity, and diabetes. It is important to recall the distinction between sex, defined by chromosomal makeup, and gender, which is a social/cultural construct. Thus, in humans, sex differences arise from gene-dosage effects of the X and Y chromosomes (8, 9), which are elicited to a great extent through the actions of sex hormones. The effect of sex chromosomes themselves on metabolism has been clearly demonstrated by analyses of the four core genotypes mouse model that demonstrated that the sex chromosome complement controls adiposity, feeding behavior, fatty liver, and systemic glucose homeostasis (9), independent of gonadal sex. The mechanisms of sex differences in central nervous system (CNS) control of energy homeostasis in health and disease have been well summarized in several recent reviews (10–14).

## Sex-Specific Differences in Fat Distribution

Women have higher percent body-fat (15, 16), less visceral white adipose tissue (V WAT), and more subcutaneous adipose tissue (SC WAT), both in the abdominal (17–20) and gluteofemoral (21, 22) regions. Various factors responsible for individual variability and sex differences in fat distribution have been described in detail in a recent review (23). Although body-mass index (BMI) is a strong predictor of overall mortality in humans (24), body-fat distribution is a stronger predictor of metabolic health. For example, low SC WAT is a favorable factor, whereas high V WAT is an unfavorable factor associated with altered lipid and glucose homeostasis (25–27). Furthermore, gluteal-femoral fat may have a protective effect against diabetes and overall mortality (28–30), suggesting that the effective compartmentalization of free fatty acids (FFAs) in low SC WAT may prevent abdominal obesity and associated metabolic disease (31). Regional differences in FFA metabolism do not fully explain sex-specific WAT distribution in humans, although a general trend in regional lipid utilization is consistent with the android and gynoid body types observed in males and females, respectively (25).

## Sex-Specific Differences in Lipid Metabolism

### Lipolysis

**Figure 1:**
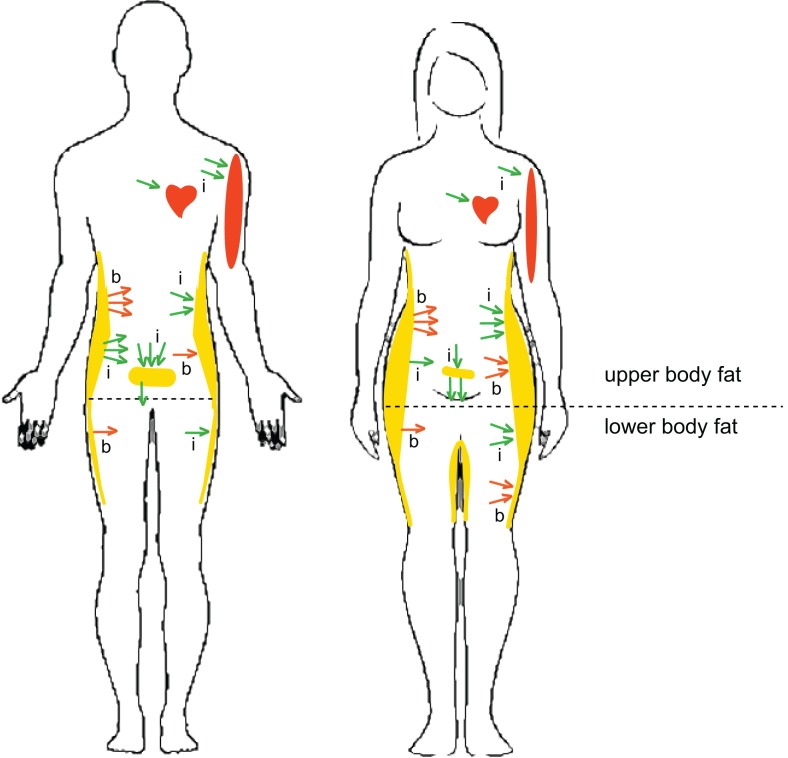
**Sex- and depot-specific differences in lipid metabolism in humans**. Women have higher percent body-fat and lower muscle mass and oxidize less lipid substrate than men. Women also have less V WAT and more SC WAT, both in the abdominal and gluteofemoral regions, than men. Outward arrows indicate basal (b) and insulin-inhibited (i) lipolysis. Inward arrows indicate basal (b) and insulin-stimulated (i) FFA uptake. Under basal post-absorptive conditions, upper-body SC WAT is more lipolytically active than lower-body SC WAT in both men and women (red outward arrows). The release of postprandial FFAs from upper-body SC WAT is less suppressed by insulin in men and FFA release from V WAT is less suppressed in women (green outward arrows). A greater percentage of postprandial FFAs are taken up by upper-body SC WAT in women than in men. Women display higher direct FFA uptake in leg SC WAT than men. Direct FFA uptake is higher in upper SC WAT than in lower SC WAT in men, but not in women. These sex differences in the topography of lipid metabolism may explain higher SC WAT, especially low-body SC WAT, in women compared to men. Higher muscle mass in males is beneficial for more efficient oxidation of FFAs.

Under basal post-absorptive conditions, systemic FFA flux is similar in men and women (32), although upper-body SC WAT is more lipolytically active than lower-body SC WAT in both men and women [Figure 1; (32, 33)]. Non-oxidative FFA clearance through re-esterification, however, is higher in women than in men (34), suggesting that women tend to store, whereas men tend to oxidize, circulating FFAs (35). Men are less sensitive to the antilipolytic effects of insulin; i.e., the release of postprandial FFAs from upper-body SC WAT is less suppressed (more insulin-resistant) in men and FFA release from V WAT is less suppressed in women (32). This pattern of lipolysis is consistent with higher proportion of V WAT found in men (Figure 1). After a meal, systemic FFA flux is more suppressed by insulin in women (32), suggesting that women may have a higher risk of fat gain. Because the contribution of V WAT lipolysis to hepatic FFA delivery is greater in women, they are also more susceptible to hepatic insulin resistance (36, 37).

### FFA uptake and triglyceride storage

Although most lipid adsorption in human WAT tissue is mediated via hydrolysis of circulating triglycerides (TG) by lipoprotein lipase (LPL), it is now recognized that direct, LPL-independent FFA uptake may also contribute to lipid storage in humans (38, 39). A greater percentage of meal FFAs is stored (primarily by the LPL-dependent pathway) in upper SC WAT than in lower SC WAT in both men and women, but a greater percentage of dietary FFAs is stored in SC WAT in women than in men, who tend to store FFAs in V WAT [Figure 1; (35, 40)]. Interestingly, testosterone treatment increases the percentage of FFAs stored in SC WAT and decreases FFA storage in V WAT in middle-aged men (41). Basal FFA uptake and expression levels of FFA transporters are higher in upper SC WAT than in lower SC WAT in men, but not in women (38, 39). Women, however, display higher FFA uptake in leg SC WAT, which correlates with greater postprandial WAT LPL activity in women [Figure 1; (42)]. Furthermore, women have the highest rate of TG synthesis in their SC WAT compared to their visceral or any WAT depot in men (43). The menstrual cycle has no apparent effect on FFA tissue uptake in healthy women, consistent with studies in female non-human primates (NHPs) (35, 44).

## Sex-Specific Differences in Glucose Metabolism and Insulin Action

Glucose metabolism in both sexes is highly responsive to physiological and nutritional states and physical fitness (45). During exercise, women oxidize more lipids and less carbohydrates, deplete less muscle glycogen, and exhibit lower hepatic glucose production (46, 47). At high altitude, women are able to attenuate the use of carbohydrates (48). This preferential substrate selection is attributed to estrogens (49). The potential mechanisms responsible for sex-specific metabolic responses to exercise include lower sympathetic nerve activity (50) and a greater type I and type II muscle fiber density (51) in women. Despite having a lower percentage of fat mass, the prevalence of type-2 diabetes and insulin resistance is higher in men (52–54). These differences are explained by higher whole-body insulin sensitivity in women (55–57). The human data are consistent with rodent studies that demonstrated greater insulin sensitivity and greater resistance to a high-fat diet (HFD) in females (58–61). The majority of human studies showed that glucose effectiveness and glucose appearance rates (62) are also higher in women (63–66).

The mechanisms responsible for sex differences in insulin sensitivity are not understood. One study reported no differences in insulin secretion between young males and young females (64), whereas others showed that females have higher first-phase insulin secretion than males (65). Sex differences in insulin signaling are tissue-specific, but have been primarily studied in rodent models (67). Kahn and colleagues reported that, in rodents, female WAT had a greater response to insulin and a greater increase in Akt and extracellular signal-related kinase (ERK) phosphorylation and lipogenesis than male WAT (61). Castration increased the insulin responsiveness of male WAT, while ovariectomy decreased insulin responsiveness of female WAT (61). Our studies in NHPs, however, suggest that, in contrast to rodents, testosterone promotes insulin-stimulated Akt phosphorylation and stimulates lipogenesis in WAT of castrated males (68). Greater rates of insulin-stimulated glucose uptake in female skeletal muscle and WAT correlate with higher expression levels of muscle mRNAs encoding glucose transporter-4 (Glut4) and metabolic enzymes (69).

## Sex Hormones and Metabolism

### Estrogens, progesterone, and menstrual cycle

The use of oral steroidal contraceptives is associated with a reduction in insulin sensitivity and low estrogen levels (70), suggesting that estrogens may protect females against insulin resistance. Consistent with this idea, studies in estrogen-deficient aromatase-knockout (ArKO) and ovariectomized wild-type mice showed that estrogen replacement can protect female mice against hepatic steatosis and improve mitochondrial β-oxidation (71) and insulin sensitivity (61, 72). The molecular mechanisms responsible for estrogen-mediated improvement in lipid-induced insulin resistance are tissue and pathway-specific and estrogen’s effects on various signal transduction and metabolic pathways in adipocytes, myocytes, and hepatocytes have been described in detail in a recent review (73).

Many anti-obesity effects of estrogens in mice are mediated by central mechanisms. Important regulators of metabolism have receptors in the CNS that mediate overall energy expenditure, food intake, glucose, and homeostasis (74). However, little is known about sex differences in CNS regulation of metabolism and most research has focused on estrogen action (75). The majority of estrogen’s anorexic action in the hypothalamus is mediated by estrogen receptor-α (ERα), and not estrogen receptor-β (ERβ) (76), although one study contested this notion (77). Diminished ERα activity is associated with obesity in both sexes. Estrogen-deficient androgen receptor (AR) knockout and ERα knockout male and female mice are obese and have decreased energy expenditure (78–80).

In addition to central mechanisms, estrogens can also directly suppress TG synthesis by reducing lipogenesis in the liver and increasing lipolysis in adipocytes (81–83). Estrogens potentiate lipid accumulation in SC WAT in males and females (84, 85), promote FFA β-oxidation, and reduce TG storage by stimulating the expression of peroxisome proliferation activator receptor-delta (PPARδ) and by activating AMP-activated protein kinase [AMPK; (81)]. In pancreas, estrogens exert protective effects on β-cell function by reducing amyloid formation in ovariectomized females (86) and obese males (87), and decrease β-cell injury induced by streptozotocin (STZ) in both sexes (88, 89). Interestingly, men lacking estrogen production or signaling because of mutations in the genes encoding aromatase or ERα are insulin-resistant and glucose-intolerant (90, 91), suggesting that estrogens may also regulate energy homeostasis in males. Although the beneficial effects of estrogens on whole-body glucose metabolism and insulin sensitivity have been demonstrated in several rodent models (73, 92), sex-specific effects in humans remain to be further elucidated.

Glucose metabolism is also affected by the menstrual phase, when the energy demand is high, which can be explained by the suppressive effect of estrogens and progesterone on gluconeogenesis (93). Estrogens promote insulin sensitivity, whereas progesterone promotes insulin resistance by antagonizing the positive effect of estrogens on contraction-stimulated glucose uptake (94) and increasing the activity of the β-oxidation pathway in skeletal muscle during exercise (95, 96). Glucose metabolism and exercise performance are influenced by the menstrual cycle phase in that glucose appearance and disappearance rates are higher during the follicular phase (high estrogen-to-progesterone ratio) compared to the luteal phase (high progesterone-to-estrogen ratio) (97). Muscle glycogen utilization depends on FFA availability in that there is an inverse correlation between FFA concentration and muscle glycogen use during exercise. The opposing actions of estrogens and progesterone on glycogen utilization may be mediated by their impact on FFA availability (98). Thus, glucose and lipid metabolism depends on the menstrual cycle phase, which determines the progesterone-to-estrogen ratio, and nutritional and exercise status.

### Androgens

A significant portion of the world population suffers from various forms of metabolic abnormalities related to androgen imbalance, including hypoandrogenism in men (99–105) and hyperandrogenism in women (99, 106). Androgen deficiency in men is associated with insulin resistance and obesity, and treatment of hypogonadal men with testosterone improves insulin sensitivity and reduces fat content (99–105). In contrast, the androgen excess observed in women with polycystic ovary syndrome (PCOS) is associated with insulin resistance and obesity (99, 106).

Sex-dependent actions of androgens in WAT may explain differences in body-fat distribution (107) and insulin sensitivity in males and females (52, 54–56, 61). Aromatization of testosterone to 17β-estradiol is important for energy homeostasis, and an increased androgen-to-estrogen ratio promotes visceral obesity in males (108, 109). Men with genetic androgen resistance due to decreased AR expression develop visceral obesity (110), and AR knockout mice demonstrate higher visceral adiposity and insulin resistance (111, 112), suggesting that androgens may suppress WAT mass both in humans and rodents. Although human studies consistently demonstrate a positive correlation between hypogonadism, increased body-fat, and insulin resistance (99–105, 113, 114), it is unknown whether these changes represent direct effects of androgen imbalance on target tissues or secondary effects of aging or changes in lifestyle on whole-body metabolism and body composition.

For example, testosterone promotes the commitment of pluoropotent mesenchymal stem cells to the myogenic lineage (115), which may indirectly improve energy expenditure and accelerate fat mass loss, contributing to improved insulin sensitivity in testosterone-supplemented males. Testosterone increases the expression of the transcription factor PGC1α in skeletal muscle (116), stimulating mitochondrial biogenesis, substrate oxidation, and muscle insulin sensitivity. Lower levels of PGC1α are found in insulin-resistant type-2 diabetic patients (117). In rodents, testosterone exerts gender-specific protective effects against STZ-induced apoptosis of male β-cells (118, 119). Interestingly, androgen administration to normally STZ-resistant female rats make them highly susceptible to β-cell death (88). Thus, in rodents testosterone is involved in a gender-specific regulation of β-cell number, playing a protective antidiabetic role in males, but can override the protective role of estrogens in females, illustrating the importance of the androgen/estrogen ratio in β-cell survival in females.

## Sex-Specific Effects in NHPs

Consistent with human studies, female rhesus macaques exhibit significantly higher levels of insulin-stimulated glucose disposal than males (120) and estrogens can improve glucose regulation in female macaques (121). In contrast to estrogen effects, adult female rhesus macaques exposed to androgens developed insulin resistance, although this effect was only apparent in animals consuming a HFD (44). Studies in prenatally androgenized monkeys further demonstrated that androgens disrupt insulin sensitivity and glucoregulation in female offspring (122, 123). These findings are consistent with the idea that exposure of females to androgens may trigger a metabolic reprograming toward a male-like phenotype.

Androgen effects in adult female NHPs are menstrual cycle-dependent, in that V WAT lipolysis and hormone-sensitive lipase (HSL) expression were upregulated during the luteal phase compared with the early follicular phase of the ovarian cycle, while hyperandrogenemia attenuated lipolysis and HSL expression during the luteal phase but not during menses (44). Insulin sensitivity of FFA uptake in WAT was also differentially affected by testosterone in a menstrual cycle-dependent manner (44). Collectively, these findings may explain the predisposition of women with PCOS to WAT dysfunction and obesity (99, 106). Androgen supplementation studies in adult male rhesus macaques suggest that androgens do not control whole-body insulin sensitivity, at least under conditions of a low-fat (chow) diet (68, 124), but a lack of testosterone can have a negative impact on WAT biogenesis and function (68). Prenatally androgenized male rhesus macaques, however, develop insulin resistance, suggesting that early exposure to androgens may cause fetal reprograming of male metabolic tissues (125). Interestingly, testosterone stimulates WAT lipogenic gene expression in both males and females (44, 68), suggesting that androgens can facilitate maturation of WAT in both sexes.

## Conclusion

Overall, female lipid metabolism functions to store more fat in SC WAT, with a higher proportion of lipids deposited in lower body-fat, while men store more lipids in V WAT than in SC WAT and oxidize dietary FFAs more readily than women. These differences presumably reflect the opposite sex-specific specialization in energy utilization, with women having the unique burden of gestation and lactation. Higher insulin sensitivity and lower muscle mass observed in women is more beneficial for energy storage in WAT and less beneficial for its oxidation. Furthermore, more efficient insulin inhibition of lipolysis facilitates greater TG retention in female WAT than in male WAT. Estrogens and androgens exert beneficial metabolic effects by lowering body-fat and improving insulin sensitivity in females and males, respectively, although the role of androgens in regulating insulin action in humans, especially under conditions of different diets, remains controversial. The use of NHP models may help separate the direct effects of sex hormones on metabolic tissues from indirect effects, such as the differences in lifestyle, diet, and prior medical history observed in human patients.

## Conflict of Interest Statement

The authors declare that the research was conducted in the absence of any commercial or financial relationships that could be construed as a potential conflict of interest.
